# Special Issue on Drug–Membrane Interactions Volume II

**DOI:** 10.3390/membranes12101018

**Published:** 2022-10-20

**Authors:** Marina Pinheiro, Sandra G. Silva

**Affiliations:** 1 LAQV, REQUIMTE, Departamento de Ciências Químicas, Faculdade de Farmácia, Universidade do Porto, 4050-313 Porto, Portugal; 2 LAQV, REQUIMTE, Departamento de Química e Bioquímica, Faculdade de Ciências, Universidade do Porto, Rua do Campo Alegre s/n, 4169-007 Porto, Portugal

There is no life without cells and there are no cells without membranes. On the other side, there would be no full knowledge of the mechanisms of the actions of a drug without a deeper understanding of its interaction with membranes. In this context, the second edition of the Special Issue on drug–membrane interactions highlights the importance of the interactions of drugs with membranes to better understand a drug’s mechanism of action at the molecular level [1]. The transport of drugs across cell membranes is a complex biological process, dynamic in nature, which is not yet completely elucidated. In this regard, simple membrane models are generally used and are associated with different biophysical techniques to allow the understanding of the mechanisms of the actions of the drugs at a big picture level from the medication intake until the drugs’ elimination [2]. Indeed, a full understanding of the drug’s pharmacokinetics and pharmacodynamics is essential to investigating drug-membrane interactions. In this Special Issue entitled “Study on Drug-Membrane Interactions, Volume II”, different contributions to the fascinating field of drug–membrane interaction studies are provided. The Special Issue contains four research articles which the editors invite the readers to explore. A brief description of each study is given bellow.

Rynkowska *et al*. evaluated the protective effect of two antioxidants, melatonin and indole-3-propionic acid (IPA), against oxidative damage to membrane lipids, induced by high concentrations of iron, in porcine skin. Both antioxidants were shown to be efficient in the reduction of lipid peroxidation. The authors concluded the two compounds are potential therapeutic agents to treat disorders associated with excessive iron accumulation in the skin [3].

Menna and co-workers studied the interaction of dendritic cells (DCs) with the Blood–brain barrier (BBB) in steady-state conditions. The authors observed that transmigrated DCs exhibit an activated phenotype and a stronger T cell-stimulatory capacity when compared to non-migrating DCs. The results obtained are of the utmost importance for the design of novel targeted therapies capable of inhibiting autoimmune inflammation of the central nervous system [4].

The study conducted by Bolla and co-workers reports the inhibitory effect of garcinol formulations in the activity of cytochrome P450 isozymes and P-glycoprotein (P-gP). Both *in vitro* and *in vivo* studies and, in some cases, molecular docking studies were performed. The findings reported allowed for the prediction of potential herb–drug/drug–drug interactions of garcinol for safe clinical use [5].

The work reported by Wang describes the effects of potassium ions on surface pressure isotherms, elastic modulus and the stability of DPPC/Cholesterol and DPPC/Cholesterol/Amphotericin B monolayers using Langmuir-Blodgett methodology [6]. The results presented contributed to the understanding of the complex mechanism of the interaction of Amphotericin B with membranes and the influence of potassium ions.

The investigation of this enthusiastic topic has just started and a long path needs to be done to develop novel drugs with even more efficacy and safety for the global health benefit.

## Data Availability

Not applicable.
